# 55715 Quantification of Neonatal THC Exposure Following Prenatal Marijuana Use

**DOI:** 10.1017/cts.2021.699

**Published:** 2021-03-30

**Authors:** Stefanie Kennon-McGill, Heather Moody, Jeff Moran, Laura James

**Affiliations:** University of Arkansas for Medical Sciences

## Abstract

ABSTRACT IMPACT: Quantification of neonatal THC exposure will allow for better insight into how THC exposure correlates with neurodevelopmental outcomes. OBJECTIVES/GOALS: Tetrahydrocannabinol (THC) use has become increasingly prevalent in recent years, including among pregnant women. However, few, if any, clinical studies have quantified precise in utero exposure levels of THC during pregnancy. Our study aims to fill this gap by using analytical methods to quantify THC in mother and baby following prenatal THC use. METHODS/STUDY POPULATION: Pregnant women were asked to give a self-report of all cannabis and cannabinoid use during pregnancy, including dose, frequency, and route of consumption. Upon arrival at the labor and delivery unit, maternal blood samples were collected. Immediately following birth and 24 hours after birth, umbilical cord and neonatal blood samples were collected, respectively. All blood samples were analyzed using tandem liquid chromatography-mass spectrometry (LC-MS) for the presence of THC, tetrahydrocannabinol carboxylic acid (THC-COOH), and hydroxy-tetrahydrocannabinol (THC-OH). Maternal THC and metabolite levels were compared to both cord and neonate samples. RESULTS/ANTICIPATED RESULTS: To date, we have collected 3 mother-infant sample dyads and 4 mother-infant control samples. We anticipate collecting a total of 20 mother-infant samples from each group. We will quantify levels of THC and its metabolites in maternal samples and compare these to cord and infant samples. We expect that THC/metabolite levels will vary as a function of dose and frequency of consumption. We also expect that THC/metabolites will be higher in umbilical cord blood relative to neonatal blood. DISCUSSION/SIGNIFICANCE OF FINDINGS: This study is among the first to directly measure exposure in the neonate following prenatal cannabis use. Quantification of THC/metabolite concentrations will be supplemented with developmental evaluations of infants at 6 and 12 months of age in order to gain better insight into how THC exposure correlates with neurodevelopmental outcomes.

